# Identification and Validation of Reference Genes for Gene Expression Analysis Using Quantitative PCR in *Spodoptera litura* (Lepidoptera: Noctuidae)

**DOI:** 10.1371/journal.pone.0068059

**Published:** 2013-07-09

**Authors:** Yanhui Lu, Miao Yuan, Xiwu Gao, Tinghao Kang, Sha Zhan, Hu Wan, Jianhong Li

**Affiliations:** 1 College of Plant Science and Technology, Huazhong Agricultural University, Wuhan, China; 2 Department of Entomology, China Agricultural University, Beijing, China; Kyushu Institute of Technology, Japan

## Abstract

Reverse transcription quantitative polymerase chain reaction (qRT-PCR) has rapidly become the most sensitive and accurate method for the quantification of gene expression. To facilitate gene expression studies and obtain more accurate qRT-PCR data, normalization relative to stable housekeeping genes is required. These housekeeping genes need to show stable expression under the given experimental conditions for the qRT-PCR results to be accurate. Unfortunately, there are no studies on the stability of housekeeping genes used in *Spodoptera litura*. In this study, eight candidate reference genes, elongation factor 1 alpha (*EF1*), glyceraldehyde-3-phosphate dehydrogenase (*GAPDH*), ribosomal protein L10 (*RPL10*), ribosomal protein S3 (*RPS3*), beta actin (*ACTB*), beta FTZ-F1 (*FTZF1*), ubiquinol-cytochrome c reductase (*UCCR*), and arginine kinase (*AK*), were evaluated for their suitability as normalization genes under different experimental conditions using the statistical software programs, BestKeeper, geNorm and Normfinder, and the comparative ΔCt method. We determined the expression levels of the candidate reference genes for three biotic factors (developmental stage, tissue and population), and four abiotic treatments (temperature, insecticide, food and starvation). The results indicated that the best sets of candidates as reference genes were as follows: *GAPDH* and *UCCR* for developmental stages; *RPL10*, *AK* and *EF1* for different tissues; *RPL10* and *EF1* for different populations in China; *GAPDH* and *EF1* for temperature-stressed larvae; *AK* and *ACTB* for larvae treated with different insecticides; *RPL10*, *GAPDH* and *UCCR* for larvae fed different diets; *RPS3* and *ACTB* for starved larvae. We believe that these results make an important contribution to gene analysis studies in *S. litura* and form the basis of further research on stable reference genes in *S. litura* and other organisms.

## Introduction

Reverse transcription quantitative polymerase chain reaction (qRT-PCR) has rapidly become the most sensitive, accurate and widely used method for gene expression analysis in order to understand biological processes and physiological functions, as well as for validation of the results of microarray analysis and other techniques [1], [2]. One of the critical challenges of qRT-PCR analysis for reliable mRNA quantification in any biological system is the availability of appropriate normalization genes, the expression level of which is considered stable, regardless of cell type and across various experimental conditions [3]. However, several studies have revealed that using different normalization genes which show variations in a biological system can result in appreciable errors owing to different treatments, sampling methods, total RNA extraction, reverse-transcription, etc., even up to 20-fold by some estimations [4]–[6]. Hence, the use of normalization genes should be experimentally validated for different developmental stages, tissues and specific experimental designs [3], [6], [7]. Furthermore, at least two or three reference genes should be used for accurate normalization based on the studies of Thellin et al. [8] and Vandesompele et al. [6]. In most studies, normalization has been described for certain systems but is frequently applied to other systems without an appropriate validation of their stability in that particular system. Therefore, it is necessary to select the most suitable genes for normalization from a panel of candidate genes in a given set of biological samples from a specific organism.

*Spodoptera litura* is an important polyphagous insect pest that causes widespread economic damage to vegetables and other crops, including ornamental plants in tropical and subtropical regions [9], [10]. As a polyphagous species, this pest has the potential to invade new areas and to adapt to new host plants. In recent years, molecular technology, particularly qRT-PCR for gene expression, has been widely used in genetic studies on *S. litura* [11], [12]. Changes in gene expression can often reflect biologically significant changes across insect developmental stages, tissues and other samples from different experimental conditions. Therefore, it is important to establish normalization genes so that specific changes in gene expression can be evaluated. However, no experimental data are available on the most appropriate normalization genes under different conditions and at different developmental stages for the sensitive detection of target gene transcripts in *S. litura*. In this study, we identified and examined eight normalization genes for *S. litura* in different developmental stages, tissues and under different treatments, in order to assess which of these genes were the most stable and therefore represented the best choice for qRT-PCR experiments on *S. litura* under different conditions. The eight selected genes were ribosomal protein S3 (*RPS3*), ribosomal protein L10 (*RPL10*), beta-actin (*ACTB*), ubiquinol-cytochrome c reductase (*UCCR*), transcription factor beta FTZ-F1 (*FTZF1*), glyceraldehyde-3-phosphate dehydrogenase (*GAPDH*), elongation factor (*EF1*) and arginine kinase (*AK*). These genes are commonly used as single normalization genes in gene expression studies of *S. litura* and other insects.

## Materials and Methods

### Insects

The laboratory strain of *S. litura* was established from field collections in June 2012 obtained from a lotus field at the Agricultural Experiment Station, Huazhong Agricultural University (Wuhan, Hubei, China). The larvae were reared on lotus leaves, and the adults were fed 10% honey solution in the laboratory. They were reared at a temperature of 30°C, under a photoperiod of 16∶8 h L: D, and relative humidity of 70%. Other populations used in this experiment were collected from lotus fields at Jiangsu, Zhejiang, Jiangxi, Anhui and Shandong provinces. The laboratory strain and other populations used in this experiment were from different fields. No specific permissions were required as these fields are experimental plots that belong to Huazhong Agricultural University, Wuhan, Hubei in China.

### Biotic Factors

*Developmental stage*: Samples used comprised 300 first-day eggs, 50 first-instar larvae, 30 second-instar larvae, five third-instar larvae, five fourth-instar larvae, five fifth-instar larvae, five sixth-instar larvae, five pre-pupae, five first-day male and female pupae, five six-day male and female pupae, five 12-day-old male and female pupae, five first-day male and female adults, and five 7-day-old male and female adults for each replication. All the samples were collected in 1.5 mL microcentrifuge tubes, which were immediately frozen in liquid nitrogen and stored at −80°C.

*Tissue*: Tissue from the brain, midgut, fat body, epidermis and hemolymph were obtained from third-instar larvae using dissection needle in PBS solution on ice [13].

*Population*: One laboratory *S. litura* strain and five field collected populations from Jiangsu, Zhejiang, Jiangxi, Anhui and Shandong provinces were used. The laboratory strain was maintained without exposure to any insecticide within the laboratory setting.

### Abiotic Stresses

#### Temperature-induced stress

Each group of five third-instar larvae were exposed to temperatures of 15°C (cold), 25°C (room temperature) or 35°C (hot) for 1 h in a glass tube placed in a water bath. Five insects in each temperature were then collected for RNA extraction.

#### Insecticide-induced stress

The insecticides used were chlorpyrifos, diafenthiuron, spinosad, indoxacarb and chlorantraniliprole, which are often used in Lepidopteran pest management programs. The leaf-dip bioassay method reported by Shelton et al. [14] and Liang et al. [15] was adopted for insecticide bioassay. Cabbage discs (6.5 cm diameter) were cut and dipped in various concentrations of insecticides prepared with distilled water containing 0.1% Triton X-100. Each disc was dipped for 10 s and allowed to air dry at room temperature. The discs were then placed individually inside plastic petri dishes (7.0 cm diameter). A total of 10–15 third-instar larvae were confined to each dish, and three replications were prepared. Controls were cabbage discs treated with distilled water containing 0.1% Triton X-100. The treated larvae were reared routinely and mortality was checked after 48 h. The 48-h LC_15_ (sublethal dose) values for the insecticides were estimated by probit analysis (Table 1). Third-instar larvae were then treated using the LC_15_ value of each insecticide. The surviving insects after 48 h were collected for RNA extraction.

**Table 1:** The toxicity of insecticides to the third-instar larvae of *S. litura*.

Insecticides	N^a^	Slope ± SE^b^	LC_15_ c	LC_50_ c	?^2^ d
Chlorpyrifos	240	1.71**±**0.20	8.08 (4.91–11.46)	32.69 (24.83–43.45)	1.38
Diafenthiuron	210	1.90**±**0.25	7.69 (5.07–10.35)	27.07 (20.74–37.56)	1.06
Spinosad	210	1.85**±**0.24	10.64 (6.05–15.41)	38.68 (29.18–50.61)	2.26
Indoxacarb	210	2.21**±**0.27	10.28 (6.40–14.18)	30.26 (23.46–38.27)	2.35
Chlorantraniliprole	210	1.63**±**0.23	0.36 (0.18–0.56)	1.58 (1.15–2.13)	0.54

a Number of tested larvae.

b SE = standard error.

c Expressed in mg/L; 95% fiducial limits (FL) of LC_15_, LC_50_ are given in parenthesis, respectively.

d Chi-square testing linearity of dose-mortality responses.

#### Food

The newly hatched larvae were reared on an artificial diet [16], lotus leaves, taro leaves or water oats, until they reached third-instar stage. Then, the third-instar larvae were collected for RNA extraction.

#### Starvation-induced stress

Thirty third-instar larvae were starved for 6 h, and were collected for RNA extraction.

### Reference Gene Selection and Primer Design

Eight commonly used reference genes were selected (Table 2). Based on the described insect reference genes in literature, the NCBI database (http://www.ncbi.nlm.nih.gov) was searched for available *S. litura* sequences: *ACTB* [17], *UCCR*, *FTZF1*, *GAPDH* [6], *EF1* [2], [3] and *AK* [18]. We explored whether *UCCR* and *FTZF1* could be used as reference genes. However, *RPS3* [1], [19] and *RPL10* (unpublished data from our laboratory) sequences were amplified based on the sequences from *Spodoptera frugiperda* and *Spodoptera exigua* (*SfRPS3*, accession no. AF429976; *SeRPL10*, accession no. EU258622). We have submitted *RPS3* (accession No., KC866374) and *RPL10* (accession No., KC866373) gene sequences from *S. litura* to GenBank. However, 28S rRNA was omitted in our study although it has been used in several qRT-PCR studies, because many literatures [3] suggested that 28S gene may not be an ideal gene for qRT-PCR due to its high expression level. All gene-specific primers were designed using Beacon Designer 8.0 software (Premier Biosoft International, Palo Alto, CA, USA; Table 2).

**Table 2:** Primer pairs used for quantitative real-time PCR.

Gene name (Abbreviation)	Accession No.	Primer Name^a^	Sequence (5′-3′)	Product length (bp)	Tm (°C)	Primer efficiency (%)	*R^2^* ^b^
Elongation factor-1	DQ192234	SlN-F1	CTCCTACATCAAGAAGATC	295	55	96.7	0.997
(*EF1*)		SlN-R1	CTTGAGGATACCAGTTTC		55		
Ribosomal protein L10	KC866373	SlN-F2	GACTTGGGTAAGAAGAAG	189	55	109.7	0.998
(*RPL10*)		SlN-R2	GATGACATGGAATGGATG		55		
Actin	DQ494753	SlN-F3	GATCATGTTTGAGACCTT	214	55	107.3	0.998
(*ACTB*)		SlN-R3	GATCTTCATGAGGTAGTC		55		
Glyceraldehyde-3-phosphate dehydrogenase	HQ012003	SlN-F4	GGGTATTCTTGACTACAC	184	55	109.6	0.996
(*GAPDH*)		SlN-R4	CTGGATGTACTTGATGAG		55		
Beta FTZ-F1	HQ260326	SlN-F5	CTGATGAGACTACACTTC	297	55	107.9	0.998
(*FTZF1*)		SlN-R5	CAGGAACTACCATTACTAG		55		
Ubiquinol-cytochrome c reductase	HQ599193	SlN-F6	GCCAAGATTGAGATCAAG	204	55	109.7	0.998
(*UCCR*)		SlN-R6	GCATACTCCGATAACTAC		55		
Ribosomal protein S3	KC866374	SlN-F7	CGGAGATCATCATTATGG	191	55	105.6	0.997
(*RPS3*)		SlN-R7	GAGTTTGTATCTGAGAGAC		55		
Arginine kinase	HQ840714	SlAK-F	CTGAAGAAGTACCTTACC	80	55	105.2	0.989
(*AK*)		SlAK-R	CAATCCAGCAGAGTTGAG		55		

a F and R refer to forward and reverse primers, respectively;

b *R* ^2^ refers to the coefficient of determination.

### Total RNA Isolation and cDNA Synthesis

Total RNA was isolated using TRIzol reagent (Invitrogen, Carlsbad, CA, USA) following the recommended procedures. The purity of all RNA samples was assessed at absorbance ratios of A260/A280 and A260/A230 with a UV-1800 spectrophotometer (Shimadzu, Kyoto, Japan), and the integrity of the RNA was immediately checked using 1.0% agarose gel electrophoresis. Then, the RNA was treated with DNaseI (Fermentas, Glen Burnie, MD, USA) according to the manufacturer’s instructions, and the first-strand cDNA template was synthesized from 1.0 µg of total RNA using the First-Strand cDNA Synthesis Kit (Fermentas) with oligo (dT)_18_ as the primer, and stored at −20°C until use the next day. Our experimental processes were consistent for all the treatments.

### Quantitative Real-time PCR

Reverse transcription quantitative PCR (qRT-PCR) was performed using SsoFast™ EvaGreen® Supermix (Bio-Rad, Hercules, CA, USA) on a Bio-Rad iQ2 Optical System (Bio-Rad) based on the method of Giulietti et al. [20]. The amplification conditions were as follows: 95°C for 30 s followed by 40 cycles of 95°C for 5 s and 55°C for 10 s. After the reaction, a melting curve analysis from 65°C to 95°C was applied to all reactions to ensure consistency and specificity of the amplified product. A 10-fold dilution series of cDNA from the whole body of five third-instar larvae was used to create the standard curve, and the qRT-PCR efficiency was determined for each gene and each treatment using the linear regression model [21]. The corresponding qRT-PCR efficiencies (E) were calculated according to the equation: E = (10^[−1/slope]^−1) × 100 [22].

### Statistical Analysis

Expression levels were determined as the number of cycles needed for the amplification to reach a fixed threshold in the exponential phase of the PCR reaction [23]. The threshold was set at 500 for all genes to determine the Ct values. Gene stabilities of the eight candidate reference genes were evaluated using the software tools BestKeeper [21], geNorm version 3.5 (http://medgen.ugent.be/~jvdesomp/genorm/) [6] and NormFinder version 0.953 (http://www.mdl.dk/publications normfinder.htm) [24]. BestKeeper uses raw data and PCR amplification efficiency to determine the best-suited standards and combines them to create an index. Ct values were converted into relative quantities and imported into the geNorm and NormFinder software programs. The geNorm algorithm first calculates an expression stability value (M) for each gene and then compares the pair-wise variation (V) of this gene with the others. Using microarray data as a training set for the algorithm, a threshold of V <0.15 was suggested for valid normalization [6]. NormFinder also ranks the stability of the tested genes independently from each other. We also used a user-friendly web-based comprehensive tool, RefFinder (http://www.leonxie.com/referencegene.php?type=reference), including the comparative ΔCt method [21], to compare and rank the tested candidate reference genes. Based on the rankings from each program, RefFinder assigns an appropriate weight to an individual gene and calculates the geometric mean of their weights for the overall final ranking. The lower ranking indicated genes with more stable gene expression.

## Results

### Total RNA Quality and PCR Amplification Efficiencies

The concentration and purity of total RNA isolated from different samples were determined using the UV-1800 spectrophotometer. The A260/A280 ratios ranged from 1.80 to 2.20 for most RNA samples, indicating a high purity of total RNA for all samples. The integrity of all total RNA samples was confirmed using 1.0% agarose gel electrophoresis.

For each of the primer pairs, the single peak qPCR melting curves suggested that each of the primer pairs amplified a unique product. The products were sequenced and showed 100% identity with the fragment sequences on which the primer design was based (Fig. S1). The linear regression coefficients (*R^2^*) of each standard curve for PCR efficiency, which was determined for each gene using 10-fold serial dilutions of the cDNA generated from third-instar larvae of the laboratory strain, were 0.989 (*AK*), 0.996 (*GAPDH*), 0.997 (*EF1* and *RPS3*) and 0.998 (*RPL10*, *ACTB*, *FTZF1* and *UCCR*) (Table 2). The PCR efficiency of the eight candidate reference genes was excellent, ranging from the lowest for *EF1* (96.7%) to the highest for *RPL10* and *UCCR* (109.7%; Table 2).

### Expression Profiles of Candidate Reference Genes

**Figure 1:**
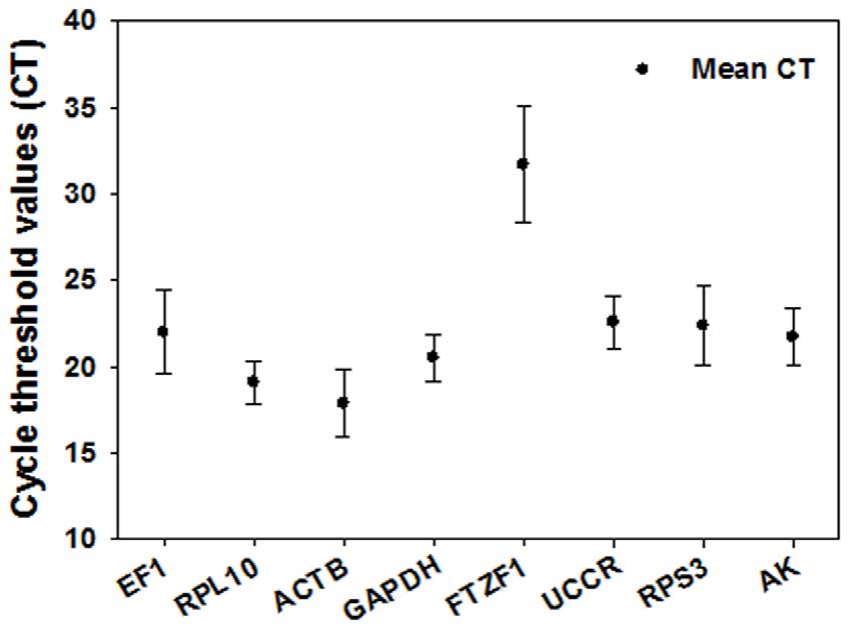
Expression levels of candidate reference genes in different samples of *S. litura*. Expression levels are displayed as cycle threshold (Ct) values of the candidate *S. litura* reference genes used in this study. The black dot indicates the mean of duplicate samples (*n* = 270), and the bars indicate the standard deviation of the mean.

Expression levels were determined as the number of cycles needed for the amplification to reach a fixed threshold (500) in the exponential phase of the PCR reaction [23]. The gene expression analysis of seven candidate reference genes, with the exception of *FTZF1,* displayed a narrow range of mean Ct values across all experimental samples (Fig. 1). The raw Ct values ranged from 14.58 (*ACTB*) to 38.59 (*FTZF1*) in all samples, from 15.92 (*ACTB*) to 33.89 (*FTZF1*) in the developmental stages, from 14.58 (*ACTB*) to 33.59 (*FTZF1*) in the different tissues, from 16.08 (*ACTB*) to 33.14 (*FTZF1*) in the different populations, from 15.58 (*ACTB*) to 35.92 (*FTZF1*) in the different temperature treatments, from 14.92 (*ACTB*) to 38.59 (*FTZF1*) in the insecticide treatments, 14.95 (*ACTB*) to 32.43 (*FTZF1*) in the third-instar larvae reared on different diets, and from 17.48 (*ACTB*) to 35.92 (*FTZF1*) in the third-instar larvae starved for 6 h. The fluorescence peak after about 15 cycles showed that *ACTB* was the most abundantly transcribed, whereas *FTZF1* was the least abundant transcript with a Ct value of 35 or higher. All candidate genes except *FTZF1* exhibited relatively small variations in Ct values.

### Analysis of Gene Expression Stability

### Biotic Factors

#### Developmental stage

**Figure 2:**
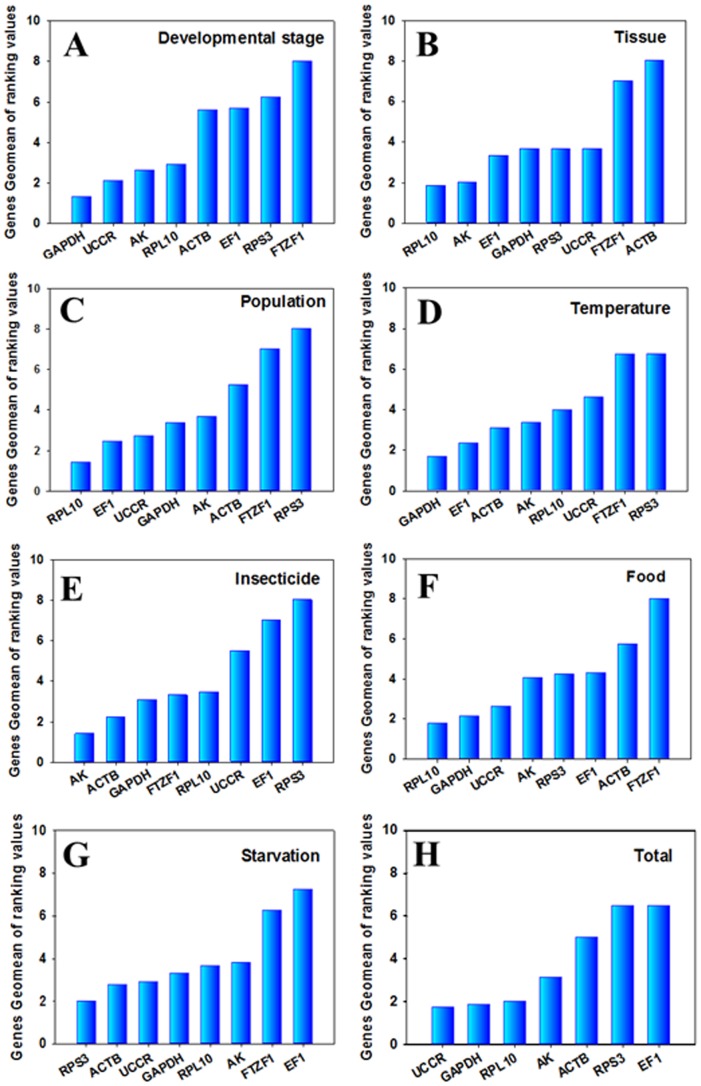
Expression stability of the candidate reference genes as calculated by the Geomean method of RefFinder (http://www.leonxie.com/referencegene.php?type=reference). A lower Geomean ranking indicates more stable expression. Expression stability of reference genes in the following samples: A) different developmental stages of *S. litura*; B) different *S. litura* tissues; C) different populations of *S. litura*; D) *S. litura* exposed to different temperatures; E) *S. litura* treated with different insecticides; F) *S. litura* fed with different diets; G) starved *S. litura*; and H) *S. litura* under all conditions.

**Figure 3:**
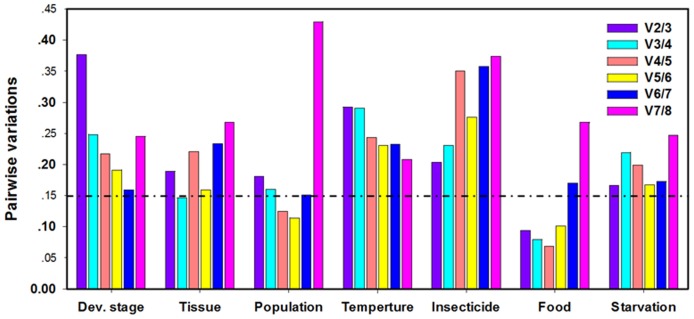
Determination of the optimal number of reference genes as calculated by geNorm for accurate normalization of gene expression. Average pairwise variations (V) were calculated by geNorm between the normalization factors NF_n_ and NF_n+1_ to indicate whether inclusion of an extra reference gene would add to the stability of the normalization factor. Values <0.15 indicate that additional genes are not required for the normalization of gene expression.

The stability rankings generated by the Delta Ct method were the same as those generated by NormFinder. Additionally, the stability rankings generated by geNorm were largely similar with the results obtained from ΔCt and Normfinder methods, even though the ranking order of the genes was different to some extent. However, the gene stability rankings by BestKeeper analysis were different to the results generated by the other three methods. All four programs, except for BestKeeper, identified *GAPDH* and *UCCR* as the most stable genes (Fig. S2). According to the results of RefFinder, the stability rankings across the developmental stages were in decreasing order of *GAPDH*, *UCCR*, *AK*, *RPL10*, *ACTB*, *EF1*, *RPS3*, and *FTZF1* (Fig. 2A). For geNorm, the V value of 0.160 obtained by the *RPS23-ACTB* pair was close to the proposed 0.15 cut-off. Moreover, the inclusion of additional reference genes did not lower the V value below the proposed 0.15 cut-off until the eighth gene was added (Fig. 3).

#### Tissue

The stability rankings generated by the Delta Ct method and NormFinder identified *RPL10* and *UCCR* as the most stable pair of genes. However, gene stability, as ranked by BestKeeper and geNorm, differed from the results generated by the ΔCt and NormFinder methods (Fig. S3). According to the results of RefFinder, the stability rankings from the most stable to the least stable gene in different tissues were *RPL10*, *AK*, *EF1*, *GAPDH*, *RPS3*, *UCCR*, *FTZF1* and *ACTB* (Fig. 2B). GeNorm analysis revealed that the pair-wise variation value V3/4 was below the proposed 0.15 cut-off (Fig. 3). An increase in variation in this value was related to a decrease in expression stability, because of the inclusion of a relatively unstable fourth gene (*GAPDH*). The inclusion of a fourth reference gene did not improve the statistical significance for each of the candidate reference gene pair groups.

#### Population

The stability rankings generated by BestKeeper and geNorm identified *RPL10* and *EF1* as the most stable pair of genes. However, gene stability as ranked by the Delta Ct method and NormFinder was different to the results generated by the BestKeeper and geNorm methods (Fig. S4). According to the RefFinder results, the stability rankings from the most stable to the least stable gene in the different populations were as follows: *RPL10*, *EF1*, *UCCR*, *GAPDH*, *AK*, *ACTB*, *FTZF1*, and *RPS3* (Fig. 2C). GeNorm analysis revealed that the pair-wise variation value V4/5 was below the proposed 0.15 cut-off (Fig. 3). This result suggests that the inclusion of a fifth reference gene would not provide any additional improvement to the statistical significance for each of the candidate reference gene pair groups.

### Abiotic Stresses

#### Temperature

All four programs, with the exception of geNorm, identified *GAPDH* as the most stable gene in the third-instar larvae treated at different temperatures. From the results of RefFinder, the stability rankings from the most stable to the least stable gene in the temperature-stressed samples were *GAPDH*, *EF1*, *ACTB*, *AK*, *RPL10*, *UCCR*, *FTZF1* and *RPS3* (Fig. 2D). However, GeNorm analysis revealed that all the pair-wise variation values were above the proposed 0.15 cut-off (Fig. 3) and identified *EF1* as the most stable gene (Fig. S5). These results indicate that normalization with three stable reference genes was required (as suggested by the geNorm manual).

#### Insecticide

All four programs except for geNorm identified *AK* as the most stable gene in the third-instar larvae treated with different insecticides (Fig. S6). According to RefFinder, the stability rankings from the most stable to the least stable in the insecticide-stressed samples were *AK*, *ACTB*, *GAPDH*, *FTZF1*, *RPL10*, *UCCR*, *EF1* and *RPS3* (Fig. 2E). However, geNorm identified *RPL10* as the most stable gene (Fig. S6). GeNorm analysis also revealed that all the pair-wise variation values were above the proposed 0.15 cut-off (Fig. 3). These results indicate that normalization with three stable reference genes was required (as suggested by the geNorm manual).

#### Food

The stability rankings generated by the Delta Ct method and geNorm identified *RPL10* and *GAPDH* as the most stable pair of genes in the third-instar larvae reared on different diets (Fig. S7). Moreover, the results determined by NormFinder also identified *RPL10* as the most stable gene. However, the stability ranking generated by BestKeeper and NormFinder identified *UCCR* as the most stable gene. Based on the RefFinder results, the stability rankings from the most stable to the least stable gene in the third-instar larvae reared on different diets were as follows: *RPL10*, *GAPDH*, *UCCR*, *AK*, *RPS3*, *EF1*, *ACTB* and *FTZF1* (Fig. 2F). GeNorm analysis revealed that the pair-wise variation value V2/3 was below the proposed 0.15 cut-off (Fig. 3). This result suggests that the inclusion of a third reference gene would not improve the statistical significance of each of the candidate reference gene pair groups.

#### Starvation

The stability rankings generated by the Delta Ct method and NormFinder identified *RPS3* and *UCCR* as the most stable pair of genes in the third-instar larvae starved for 6 h (Fig. S8). However, BestKeeper and geNorm identified *RPL10* and *ACTB* as the most stable genes, respectively. According to RefFinder, the stability rankings from the most stable to the least stable in the third-instar larvae starved for 6 h were *RPS3*, *ACTB*, *UCCR*, *GAPDH*, *RPL10*, *AK*, *FTZF1* and *EF1* (Fig. 2G). GeNorm analysis revealed that all the pair-wise variation values were above the proposed 0.15 cut-off (Fig. 3). These results indicate that normalization with three stable reference genes was required (as suggested by the geNorm manual).

#### Total

We identified the ranking of *S. litura* reference genes across all of the investigated treatments. According to RefFinder, the stability rankings from the most stable to the least stable across the different developmental stages, tissues, populations, and stressors were as follows: *UCCR*, *GAPDH*, *RPL10*, *AK*, *ACTB*, *RPS3* and *EF1* (Fig. 2H).

## Discussion

This study was conducted to identify the optimal reference genes for gene expression analyses of *S. litura* for studies of different developmental stages, tissues and abiotic stress conditions. The behaviors of eight candidate reference genes were analyzed using qRT-PCR studies. To our knowledge, this is the first study to evaluate the expression stability of different candidate reference genes for qRT-PCR in *S. litura*.

A major conclusion of this study is that few, if any, universally suitable reference genes in *S. litura* can be used for qRT-PCR analyses under various experimental conditions, as the candidate reference genes showed too much variation in expression among the different treatments (Table 3). *RPL10* exhibited the most stable expression in different tissues, populations, and in the third-instar larvae reared on different diets. *GAPDH* displayed the most stable expression in different developmental stages and in samples treated at different temperatures. *AK* and *RPS3* showed the most stable expression in samples treated with insecticides and starved for 6 h. These results indicate that the stability of reference gene expression in *S. litura* needs be investigated for each experimental treatment. These results were similar to the reference gene analysis of *Drosophila* [3].

**Table 3:** Preferable reference genes across different experimental conditions according to the software analysis.

Experimental conditions		Preferable reference genes		
Biotic factors	Developmental stage	*GAPDH*	*UCCR*	
	Tissue	*RPL10*	*AK*	*EF1*
	Population	*RPL10*	*EF1*	
Abiotic factors	Temperature	*GAPDH*	*EF1*	
	Insecticide	*AK*	*ACTB*	
	Food	*RPL10*	*GAPDH*	*UCCR*
	Starvation	*RPS3*	*ACTB*	

*GAPDH* plays a role in energy metabolism and is frequently used as a reference gene [25]. In this study, it was found to be the most stably expressed gene in the different developmental stages and temperature-stressed larvae, and the second most stable gene in the larvae fed on different foods. Scharlaken [26] also identified *GAPDH* as the most stable gene in the head of the honeybee after bacterial challenge. However, several studies have demonstrated that the stability of *GAPDH* expression was low in certain conditions, such as certain life stages of *Tetranychus cinnabarinus*, in the labial gland and fat body in *Bombus terrestris* and *Bombus lucorum*, and in the salivary glands after *Trypanosoma cruzi* injection [17], [18], [25]. These results further suggest that the expression stability of reference genes is affected by different experimental conditions.

The *RPL10* gene showed the most stable expression in the tissues, different populations, and in the larvae fed on different diets, whereas *RPS3* exhibited the most stable expression in the larvae starved for 6 h. The *RPL10* gene encodes a ribosomal protein that is a component of the 60S subunit, whereas the *RPS3* gene encodes a ribosomal protein that is a component of the 40S subunit where it forms part of the domain in which translation is initiated. The genes for various ribosomal proteins have been validated as normalization genes for qRT-PCR in many organisms, and these genes have also been reported to show the most stable expression in *Tetranychus cinnabarinus* (*RPS18*: [25]), *Apis mellifera* (*RPS18*: [26]), *Rhodnius prolixus* (*RPS18*: [27]), *Cimex lectularius* (*RPL18*: [28]), and *Schistocerca gregaria* (*RP49*: [29]). Although the genes for ribosomal proteins, including *RPS3*, *RPL10*, *RPS6* and *RPS18*, were the most stable normalization genes for broad-scale gene expression analysis in most organisms, their stability ranking was dependent upon the instrument as well as the analysis program [1].

*AK*, which is the only phosphagen kinase in two major invertebrate groups, arthropods and mollusks, was the most stable gene in larvae treated with different insecticides, and the second most stable gene in tissue samples collected from third-instar larvae. Unfortunately, it has rarely been used as a reference gene in previous studies, with the exception of a single study that identified *AK* as the most stable gene in the labial gland and fat body of *Bombus terrestris* [18].

*EF1*, which plays an important role in translation by catalyzing the GTP-dependent binding of aminoacyl-tRNA to the acceptor site of the ribosome [3], exhibited the second most stable expression in the different populations and temperature-stressed larvae, and third most stable expression in the tissue samples. Our results were in accordance with reference gene analysis in *Drosophila* [3], *Orthoptera* [30] and *Hymenoptera* [18], in which *EF1* was also identified as the most stable gene.

*ACTB*, which participates in many important cellular processes including muscle contraction, cell motility, cell division and cytokinesis, showed the second most stable expression in the temperature-stressed larvae and starved larvae, and a low stability in other treatments, including different developmental stages and tissues. It is not surprising that its transcript level varies among developmental stages and different cell types, as it has functions in various cellular processes.

In addition, *UCCR*, which plays a critical role in biochemical generation of adenosine-5′-triphosphate, has rarely been used as a reference gene in previous studies; in our study, it displayed the second and third most stable expression in the developmental stages and larvae fed on different diets.

*FTZF1* was not a suitable reference gene in all experimental conditions owing to its higher Ct values and lower stability. These results emphasize that it is necessary to optimize normalization genes in all qRT-PCR experiments under different experimental conditions.

Our data reveal the optimal number of reference genes in different experimental conditions as calculated by geNorm, which determines the pairwise variations (V) in normalization factors (the geometric mean of multiple reference genes) using n or n +1 reference genes. When several reference genes are used simultaneously in a given experiment, the probability of biased normalization decreases. In our studies, V values for the developmental stages, temperatures, insecticides and starvation were higher than the threshold value (0.15); therefore, we propose that two or three of the best reference genes should be used to obtain accurate and reliable results under these conditions, because the manual of the geNorm software (2007) suggests that the 0.15 value must not be taken as a too strict cut-off.

In agreement with our results, increasing numbers of studies in recent years have clearly demonstrated that no single gene is expressed stably in all cell types and under all experimental conditions [3]. Therefore, the expression stability of a putative reference gene needs to be verified before starting each qRT-PCR experiment. In this study, we identified several optimal sets of genes that are suitable for qRT-PCR data normalization in *S. litura* under different biotic and abiotic stress conditions: *GAPDH* and *UCCR* for developmental stages; *RPL10*, *AK* and *EF1* for the tissues dissected from third-instar larvae; *RPL10* and *EF1* for populations collected from different provinces in China; *GAPDH* and *EF1* for temperature-stressed larvae; *AK* and *ACTB* for larvae treated with different insecticides; *RPL10*, *GAPDH* and *UCCR* for larvae fed different diets; *RPS3* and *ACTB* for starved larvae. However, we did not find that the best-ranked reference genes were identical among treatments based on the analysis results of various software programs. To date, several strategies have recently been developed to evaluate the suitability of candidate reference genes as normalization genes in qRT-PCR gene expression quantification experiments, including BestKeeper, geNorm, NormFinder and RefFinder. A comparison of the rankings produced by the different approaches revealed important discrepancies, which appear to be caused by the differences between the algorithms used and their different sensitivities toward co-regulated reference gene candidates [3]. However, the analysis software programs can only suggest which genes are more suitable and the appropriate reference genes should be selected based on the experimental conditions. Therefore, each experimental investigation should establish which two or three reference genes are the most appropriate for the specific conditions (such as the developmental stages, tissues and populations) under investigation.

## Supporting Information

Figure S1 **Primer positions and amplicon sequences used for qRT-PCR.** The DNA sequences are shown from the 5′ to 3′ end, and the primer positions are shaded. The products were first amplified by regular PCR and then sent to Invitrogen for sequencing.
(TIF)

Figure S2 **Expression stability of the candidate reference genes across different developmental stages of** ***S. litura*** **.** The expression stability of the reference genes in *S. litura* across developmental stages was measured using the ΔCt method, BestKeeper, NormFinder and geNorm. A lower average stability value indicates more stable expression.
(TIF)

Figure S3 **Expression stability of the candidate reference genes in different tissues of** ***S. litura*** **.** The expression stability of the reference genes in the different tissues of *S. litura* was also measured using the ΔCt method, BestKeeper, NormFinder and geNorm. A lower average stability value indicates more stable expression.
(TIF)

Figure S4 **Expression stability of the candidate reference genes in different populations of** ***S. litura*** **.** The expression stability of the reference genes in the different populations of *S. litura* was also measured using the ΔCt method, BestKeeper, NormFinder and geNorm. A lower average stability value indicates more stable expression.
(TIF)

Figure S5 **Expression stability of the candidate reference genes in temperature-stressed samples of** ***S. litura*** **.** The expression stability of the reference genes in the temperature-stressed samples of *S. litura* was also measured using the ΔCt method, BestKeeper, NormFinder and geNorm. A lower average stability value indicates more stable expression.
(TIF)

Figure S6 **Expression stability of the candidate reference genes in insecticide-stressed samples of** ***S. litura*** **.** The expression stability of the reference genes in the insecticide-stressed samples of *S. litura* was also measured using the ΔCt method, BestKeeper, NormFinder and geNorm. A lower average stability value indicates more stable expression.
(TIF)

Figure S7 **Expression stability of the candidate reference genes in different food-reared samples of** ***S. litura*** **.** The expression stability of the reference genes in the different food-reared samples of *S. litura* was also measured using the ΔCt method, BestKeeper, NormFinder and geNorm. A lower average stability value indicates more stable expression.
(TIF)

Figure S8 **Expression stability of the candidate reference genes in starvation-stressed samples of** ***S. litura*** **.** The expression stability of the reference genes in the starvation-stressed samples of *S. litura* was also measured using the ΔCt method, BestKeeper, NormFinder and geNorm. A lower average stability value indicates more stable expression.
(TIF)
