# Supplementary figures and images for: Identification and Validation of Reference Genes for Gene Expression Analysis Using Quantitative PCR in Spodoptera litura (Lepidoptera: Noctuidae)
Source: PLoS One. 2013 Jul 9;8(7):e68059. doi: 10.1371/journal.pone.0068059 (PMC3706614; doi:10.1371/journal.pone.0068059)

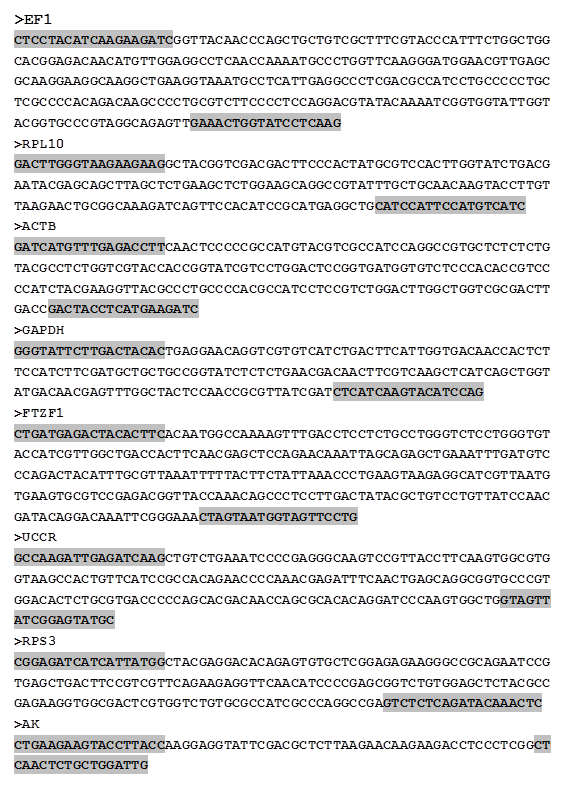

Supplement: Figure S1 — Primer positions and amplicon sequences used for qRT-PCR. The DNA sequences are shown from the 5′ to 3′ end, and the primer positions are shaded. The products were first amplified by regular PCR and then sent to Invitrogen for sequencing. (TIF) [file pone.0068059.s001.tif]

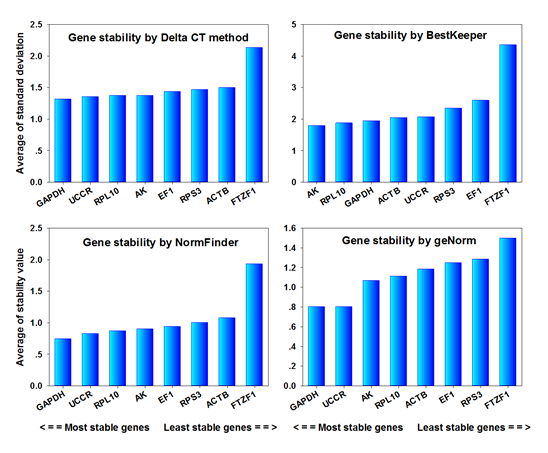

Supplement: Figure S2 — Expression stability of the candidate reference genes across different developmental stages of S. litura . The expression stability of the reference genes in S. litura across developmental stages was measured using the ΔCt method, BestKeeper, NormFinder and geNorm. A lower average stability value indicates more stable expression. (TIF) [file pone.0068059.s002.tif]

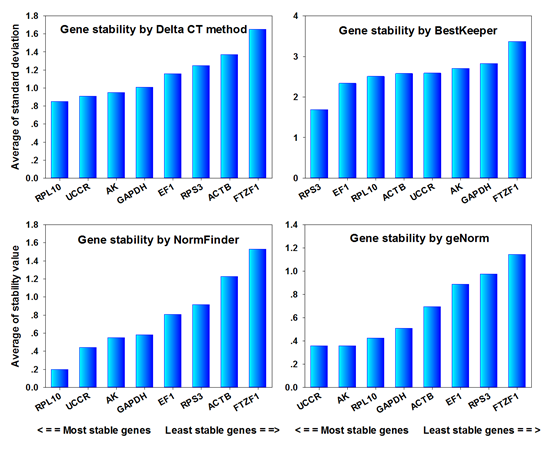

Supplement: Figure S3 — Expression stability of the candidate reference genes in different tissues of S. litura . The expression stability of the reference genes in the different tissues of S. litura was also measured using the ΔCt method, BestKeeper, NormFinder and geNorm. A lower average stability value indicates more stable expression. (TIF) [file pone.0068059.s003.tif]

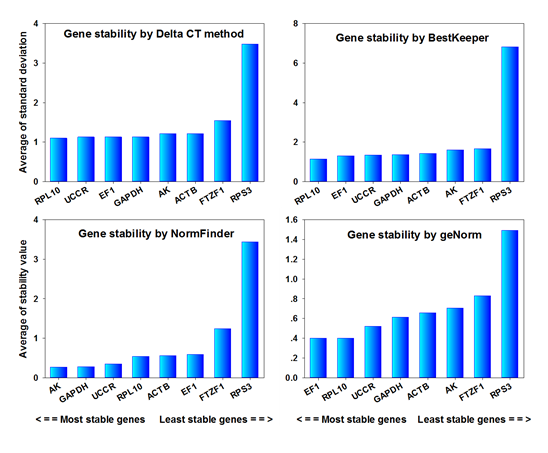

Supplement: Figure S4 — Expression stability of the candidate reference genes in different populations of S. litura . The expression stability of the reference genes in the different populations of S. litura was also measured using the ΔCt method, BestKeeper, NormFinder and geNorm. A lower average stability value indicates more stable expression. (TIF) [file pone.0068059.s004.tif]

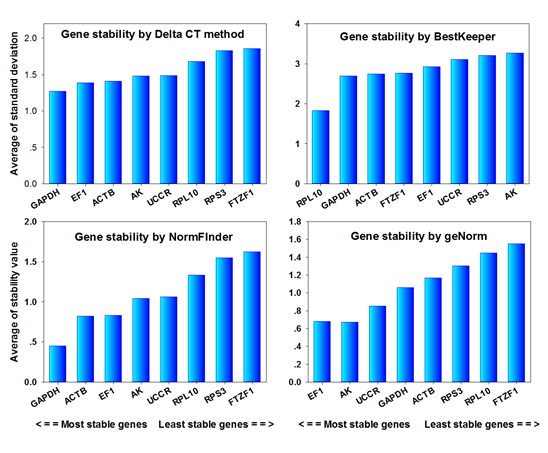

Supplement: Figure S5 — Expression stability of the candidate reference genes in temperature-stressed samples of S. litura . The expression stability of the reference genes in the temperature-stressed samples of S. litura was also measured using the ΔCt method, BestKeeper, NormFinder and geNorm. A lower average stability value indicates more stable expression. (TIF) [file pone.0068059.s005.tif]

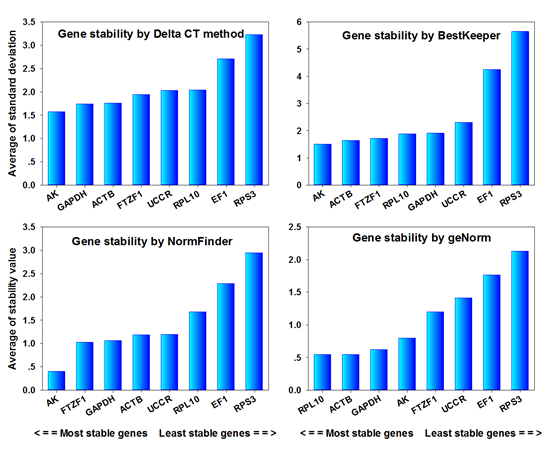

Supplement: Figure S6 — Expression stability of the candidate reference genes in insecticide-stressed samples of S. litura . The expression stability of the reference genes in the insecticide-stressed samples of S. litura was also measured using the ΔCt method, BestKeeper, NormFinder and geNorm. A lower average stability value indicates more stable expression. (TIF) [file pone.0068059.s006.tif]

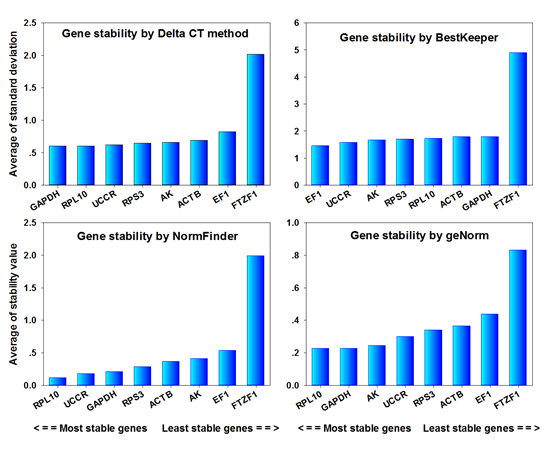

Supplement: Figure S7 — Expression stability of the candidate reference genes in different food-reared samples of S. litura . The expression stability of the reference genes in the different food-reared samples of S. litura was also measured using the ΔCt method, BestKeeper, NormFinder and geNorm. A lower average stability value indicates more stable expression. (TIF) [file pone.0068059.s007.tif]

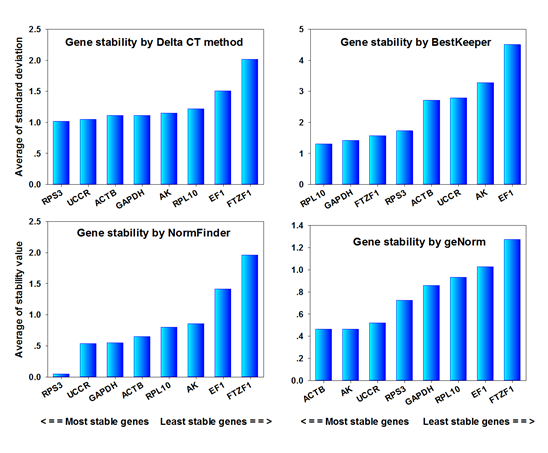

Supplement: Figure S8 — Expression stability of the candidate reference genes in starvation-stressed samples of S. litura . The expression stability of the reference genes in the starvation-stressed samples of S. litura was also measured using the ΔCt method, BestKeeper, NormFinder and geNorm. A lower average stability value indicates more stable expression. (TIF) [file pone.0068059.s008.tif]
